# A Case Where Transfusion of Blood Was Successfully Employed after Uterine Hemorrhage

**Published:** 1827-07

**Authors:** Douglas Fox

**Affiliations:** Derby, Member of the Royal College of Surgeons.


### *A Case where Transfusion of Blood was successfully employed after Uterine Hemorrhage.* By Mr. DOUGLAS FOX, of Derby, Member of the Royal College of Surgeons.

FEELING the vast importance attached to every remedy calculated to relieve the dreadful consequences attendant upon uterine hemorrhage, I am anxious to add to the general fund of information on that subject, by relating the following successful case of transfusion.

Towards the latter end of April, 1827, F. G—, ætatis thirty, living near this town, having advanced to the sixth month of pregnancy, was rather suddenly seized with labour-pains, which terminated in the expulsion of the fœtus. In a short time considerable hemorrhage commenced, in consequence of which I was requested to attend immediately. On examining into the state of the case, I found the patient extremely exhausted by great loss of blood, the pulse very feeble; that she was almost in a state of syncope, and scarcely able to speak or move. Upon inquiry, I

found the placenta was still retained. From these circumstances, it was evidently necessary to employ the most vigorous exertions to check the flow of blood, and to support the sinking powers of the patient.

Having directed that laudanum, with some stimulant, should be given, and cold water applied plentifully to the lower part of the abdomen, I introduced my hand into the uterus, which I found to be in a state of hour-glass contraction, the greater part of the placenta being in the upper cavity. Having passed my hand through the contracted portion, I discovered that the placenta was almost entirely detached, and that the hemorrhage continued to an alarming extent; but which was arrested by irritating moderately the internal surface of the uterus with the hand, and making pressure externally upon that viscus. It was some minutes before any contractile effort of the uterus was perceptible, although these means were unremittingly employed.

But, notwithstanding the hemorrhage had ceased, there was every reason to apprehend the immediate dissolution of the patient from extreme exhaustion, as in this stage of the case she had become totally unable to articulate, to move, or to swallow any kind of support: the pulse at the wrist was imperceptible, and the general appearance of the patient wore the aspect of death.

From the almost hopeless state of the case, I thought the only chance to save life was to inject blood into the system, as recommended by Dr. Blundell. Having procured my instrument for transfusion, I had one of the persons in attendance bled from a vein by my assistant; and, as the blood flowed, I injected it into the vein of the patient, previously punctured in the bend of the arm. The blood passed with facility, until about a common teacupful was injected, which occupied about six minutes. Having accomplished this, I ascertained that the pulse, which before the operation could not be distinguished, had become considerably restored; and in a few minutes more the patient was able to move without much difficulty, to speak distinctly, and to swallow any fluid food or medicine which was offered. At the same time she expressed herself to be greatly relieved; and, in short, before ten minutes had elapsed, it was evident, from the increased arterial action, the vital energy had so far returned, that, unless some untoward occurrence took place, there was every reason to anticipate a perfect recovery.

It may be well to observe, that, although every effort in this instance had been made, without any salutary effect being produced, to rally and support the powers of the patient, they had been uninterruptedly sinking from the time the hemorrhage first commenced till the transfusion was had recourse to, being an interval of upwards of an hour; and, consequently, that it was not a resuscitation from a temporary state of fainting, but from that condition which, in all probability, would have speedily proved fatal.

It is unnecessary to relate the treatment pursued after the transfusion was completed, being the same as that usually employed in order to remove the debility attendant on extensive uterine hemorrhage.

The patient has continued to improve daily, and is now recovered, being nearly three weeks since the hemorrhage took place.

*May*, 1827.

